# Tumor‐associated lymphocytes and macrophages are related to stromal elastosis and vascular invasion in breast cancer

**DOI:** 10.1002/cjp2.226

**Published:** 2021-06-02

**Authors:** Ying Chen, Tor Audun Klingen, Hans Aas, Elisabeth Wik, Lars A Akslen

**Affiliations:** ^1^ Centre for Cancer Biomarkers CCBIO, Department of Clinical Medicine University of Bergen Bergen Norway; ^2^ Department of Pathology Vestfold Hospital Tønsberg Norway; ^3^ Department of Pathology Oslo University Hospital Oslo Norway; ^4^ Department of Surgery Vestfold Hospital Tønsberg Norway; ^5^ Department of Pathology Haukeland University Hospital Bergen Norway

**Keywords:** breast cancer, tumor‐infiltrating lymphocytes, tumor‐associated macrophages, vascular invasion, stromal elastosis, mammography screening, prognosis

## Abstract

The tumor microenvironment plays a critical role in breast cancer progression. Here, we investigated tumor‐infiltrating lymphocytes (TILs) and associations with macrophage numbers, tumor stromal elastosis, vascular invasion, and tumor detection mode. We performed a population‐based retrospective study using data from The Norwegian Breast Cancer Screening Program in Vestfold County (2004–2009), including 200 screen‐detected and 82 interval cancers. The number of TILs (CD45+, CD3+, CD4+, CD8+, and FOXP3+) and tumor‐associated macrophages (CD163+) was counted using immunohistochemistry on tissue microarray slides. Lymphatic and blood vessel invasion (LVI and BVI) were recorded using D2‐40 and CD31 staining, and the amount of elastosis (high/low) was determined on regular HE‐stained slides. High numbers of all TIL subsets were associated with LVI (*p* ≤ 0.04 for all), and high counts of several TIL subgroups (CD8+, CD45+, and FOXP3+) were associated with BVI (*p* ≤ 0.04 for all). Increased levels of all TIL subsets, except CD4+, were associated with estrogen receptor‐negative tumors (*p* < 0.001) and high tumor cell proliferation by Ki67 (*p* < 0.001). Furthermore, high levels of all TIL subsets were associated with high macrophage counts (*p* < 0.001) and low‐grade stromal elastosis (*p* ≤ 0.02). High counts of CD3+, CD8+, and FOXP3+ TILs were associated with interval detected tumors (*p* ≤ 0.04 for all). Finally, in the luminal A subgroup, high levels of CD3+ and FOXP3+ TILs were associated with shorter recurrence‐free survival, and high counts of FOXP3+ were linked to reduced breast cancer‐specific survival. In conclusion, higher levels of different TIL subsets were associated with stromal features such as high macrophage counts (CD163+), presence of vascular invasion, absence of stromal elastosis, as well as increased tumor cell proliferation and interval detection mode. Our findings support a link between immune cells and vascular invasion in more aggressive breast cancer. Notably, presence of TIL subsets showed prognostic value within the luminal A category.

## Introduction

Mammography screening identifies a subset of breast cancers with a better prognosis than expected based on standard histopathologic and molecular prognostic features [1, 2, 3]. There are challenges of overdiagnosis and overtreatment following screening [4, 5, 6, 7], and new biomarkers are needed to better guide and improve therapies toward a successful clinical outcome, and to avoid overtreatment.

Breast tumors are composed of a heterogeneous population of cells including both tumor cells and the surrounding microenvironment. Stromal components facilitate cancer progression and can be predictive of both prognosis and treatment response [8, 9, 10]. Increased knowledge of the tumor microenvironment of screen‐detected breast cancers is needed to possibly explain their indolent behavior and good prognosis in most of the cases.

Previously, we reported that deposition of abnormal elastin material (elastosis) in breast cancer stroma is associated with mammographic screen detection, low tumor cell proliferation by Ki67 expression, and a favorable patient prognosis [11]. In a subsequent study, we found that tumor cell invasion into blood vessels strongly correlates with interval detected breast cancers and a basal‐like tumor phenotype [12], as well as low stromal elastosis. Also, tumor‐associated macrophages (TAM) expressing CD163 were related to vascular invasion, nonluminal subtypes, and interval breast cancer [13].

Based on the importance of the tumor immune microenvironment (TIME) in breast cancer [14, 15, 16, 17, 18, 19], the purpose of our present study was to investigate the relationship between tumor‐infiltrating lymphocytes (TILs) and tissue macrophages with the presence of vascular invasion and stromal elastosis in breast cancers stratified by detection mode, as these relationships may be of potential prognostic value and relevant for treatment strategies. The study was based on material from the population‐based Norwegian Breast Cancer Screening Program.

## Materials and methods

### Study population

A population‐based cohort of 285 patients from Vestfold County in Eastern Norway, initially including 202 invasive screen‐detected cancers and 83 invasive interval tumors, was identified. The Norwegian Breast Cancer Screening Program, implemented in 2004, involves biannual mammography in the age‐group 50–69 years. An interval cancer was defined as a cancer diagnosed between two screening sessions. Three patients were excluded: one patient with no residual tumor tissue for analysis, one screen‐detected malignant phyllodes tumor, and one patient with an interval cancer unsuitable for further surgical treatment. Four patients, two screening and two interval cancers, had simultaneous tumors in both breasts. Tumors with the worst prognostic profile, based on the Nottingham Prognostic Index, were selected for inclusion in the study cohort. Finally, 200 screen‐detected and 82 interval cancers were included for further analyses.

Information on clinical data, tumor stage, and survival was retrieved from patient records. Last clinical follow‐up was in August 2018. Regarding primary treatment, 204 breast resections (72%) and 78 mastectomies (28%) were performed; 218 patients received radiation therapy (77%), 156 received endocrine treatment (55%), and 77 received chemotherapy (27%); 12 patients lacked information on nonsurgical primary treatment. Subsequent distant metastases were observed in 42 cases (15%), and 32 patients (11%) died of breast cancer during the follow‐up period. The study was approved by the Regional Ethics Committee of Eastern Norway (reference #2018/1102).

### Tissue specimens

Three tissue cores (diameter 1.0 mm) were extracted from the invasive edge of tumor samples in paraffin‐embedded blocks and inserted into tissue microarray (TMA) recipient blocks using a semi‐automated precision instrument (Minicore 3 Tissue Arrayer; Alphelys, Plaisir, France). As reported in previous studies [13, 20, 21], TMA cores were available for 229 (81%) out of 282 cases. In 40 cases (14%), TMA cores had very limited tissue for evaluation, and whole sections were used in these cases. In 13 cases (5%), only core needle biopsies were available and used. Vascular invasion, identified by using immunostaining for CD31 and D2‐40, and presence of stromal elastosis (high/low) were determined on whole slides, and results from previous studies were included for further association analyses [12].

### Immunohistochemistry

Immunohistochemistry was performed on 4–5 μm standard tumor tissue sections of formalin‐fixed paraffin‐embedded TMAs. All slides were dewaxed with xylene/ethanol before antigen retrieval in a pressure cooker (Decloaking Chamber Plus, Biocare Medical, Concord, CA, USA), or in a microwave, in Target Retrieval Solution (TRS) buffer (pH 9) (S2367; DAKO/Agilent). An endogenous block was applied prior to incubation with the primary antibodies to block for endogenous enzymatic activity. The slides were then incubated for 1 h with mouse monoclonal CD3, CD4, CD8, CD45, and FOXP3 antibodies (A0452 [Dako/Agilent, Santa Clara, CA, USA], 104‐R15 [Cell Marque, Darmstadt, Germany], M7103 [Dako], M0701 [Dako], and M560044 [BD Biosciences, San Jose, CA, USA], respectively) followed by incubation with an appropriate HRP‐En Vision (Dako), EnVision Mouse hrp, K4001 for CD8 and FOXP3, EnVision rabbit HRP, and K4003 for CD3 and CD4. Primary antibodies were omitted for the negative controls. Tissue from differentiated breast cancer types were used as positive controls.

#### Evaluation of staining

**Figure 1:**
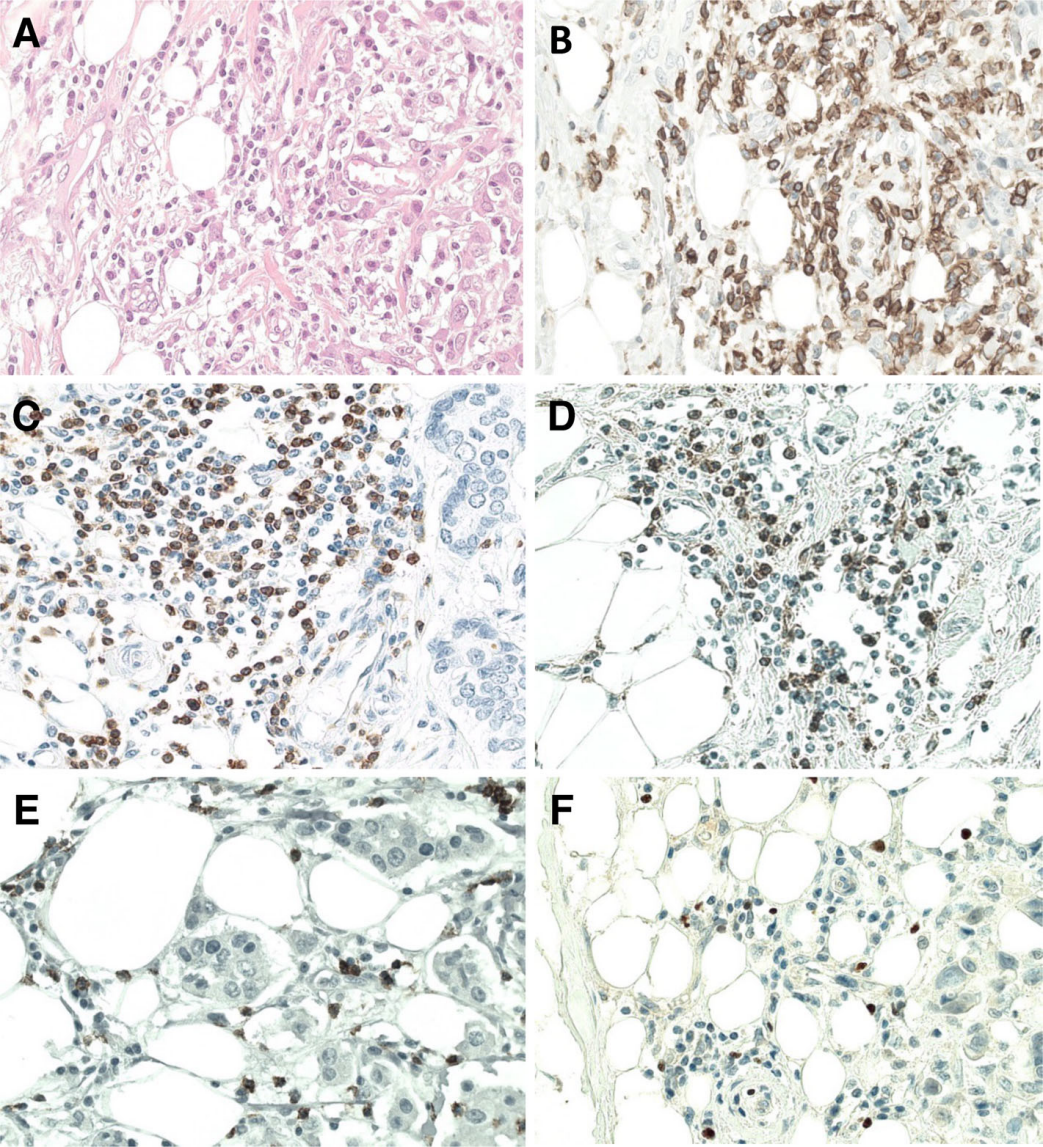
Histologic images of TIL subsets at the tumor margin by immunohistochemistry (×400). (A) HE stain, (B) CD45, (C) CD3, (D) CD4, (E) CD8, and (F) FOXP3.

Cytoplasmic CD3, CD4, CD8, CD45, and FOXP3 staining was evaluated in a quantitative manner. Staining was examined in the most active areas (hot‐spots), often situated in the periphery of the tumor (Figure 1). Lymphocytes were most often observed within stromal bands, and less often between tumor cells. The target area was evaluated by using an eye‐piece graticule (10 × 10 grid‐lines; 0.31 × 0.31 mm; total 0.096 mm^2^), and the numbers of positive cells were counted within one such area per case, similar to what we reported previously for macrophages [13].

#### Interobserver variability

A total of 50 immunostained cases were examined by two pathologists (YC and TAK), with kappa values ranging from 0.69 to 0.88 for various lymphocyte populations.

Immunohistochemical evaluations of CD163 staining for TAMs, D2‐40 staining for lymphatic vessel invasion (LVI), and CD31 staining for blood vessel invasion (BVI) have been described previously, and data were included for comparisons [12, 13]. Evaluations of HER2 status and of Ki67, estrogen receptor (ER), and progesterone receptor (PR) staining have also been reported [11, 12, 13].

### Histopathologic data

All cases were microscopically reexamined by one of the authors (TAK) using hematoxylin–eosin–safran (HES)‐stained sections from our previous study. Tumors were classified as either invasive carcinoma of no special type or other types. Histologic grading was performed according to the Nottingham criteria [22]. Tumor diameter was measured microscopically in millimeters, and lymph node status was included from pathology reports. Surrogate criteria for breast cancer molecular subtypes were applied according to the St Gallen Consensus Conference from 2013 [23]. The cut‐off point used for ER and PR was 1% using American Society of Clinical Oncology/College of American Pathologists (ASCO/CAP) guideline recommendations [24].

Elastosis can easily be recognized in standard HES slides as a deposit of gray, fibrillary material, and the histologic evaluation of tumor elastosis was described in our previous paper [11]. In brief, the amount of elastotic stroma in the tumor was evaluated, in cases with whole tissue sections available, and was graded semiquantitatively, from 0 to 3, using the criteria by Shivas and Douglas [25]. In the present study, we used data from HES‐stained tissue, and elastosis was morphologically divided into two categories, low or high; low‐grade cases had no or limited to moderate elastosis (grade 0–2) and high‐grade cases had extensive elastosis (grade 3), as reported in our previous study [11].

### Statistical analysis

All statistical analyses were performed using IBM SPSS Statistics, version 23.0 (IBM Corp., Armonk, NY, USA). Two‐sided *P* values of <0.05 were considered statistically significant. Associations between categorical variables were assessed by Pearson's chi‐square test. Spearman's rank correlation test was applied when comparing bivariate continuous variables and Spearman's correlation coefficients are reported. When analyzing differences in TIL subsets between phenotypic categories, Kruskal–Wallis test was applied. Univariate survival analysis of time to death due to breast cancer (disease‐specific survival, DSS) and time to recurrence for patients without metastases at the time of diagnosis (recurrence‐free survival, RFS) were performed using the Kaplan–Meier method (log‐rank test for differences). Entry date was the time of diagnosis. Patients who died from other causes were censored at the date of death in the analyses of DSS. A multivariable logistic regression analysis was applied to assess the ability of different TIL subsets to predict aggressive breast cancers (enter method, where all selected variables were included in one step). For statistical analysis, TIL scores were dichotomized; high TIL score (upper quartile) and low TIL score (others). A total of 282 patients were available for survival analysis in the current study.

## Results

### Presence of TIL subsets in breast cancer

Figure 1 shows lymphocyte infiltration in tumor tissues with sections, from the tumor margins, stained immunohistochemically for CD3, CD4, CD8, CD45, and FOXP3. There was very good concordance between overall lymphocyte counts on HE‐stained and CD45‐stained sections (*r* = 0.80, *p* < 0.001). Median counts of CD3, CD4, CD8, CD45, and FOXP3 are 37, 15, 18, 42, and 4, respectively (see supplementary material, Table S1). Cross‐correlation between different TIL subsets and tissue macrophages showed that they are significantly related (*p* < 0.01) (see supplementary material, Table S2).

### High levels of TIL subsets are associated with TAM counts, ER, HER2, Ki67, stromal elastosis, and breast cancer detection mode

Table 1 presents the relationships between counts of TIL subsets (CD3, CD4, CD8, CD45, and FOXP3) and clinicopathologic features, proliferation by Ki67, vessel invasion, CD163‐positive TAMs, elastosis, and tumor detection method, showing multiple significant associations. High levels of all TIL subsets in hot‐spot areas (except CD4) were strongly associated with ER negativity, HER2‐positive tumors, and high tumor cell proliferation (by Ki67). Furthermore, high levels of all TIL subsets were associated with low stromal elastosis. High levels of the T‐cell markers CD3, CD8, and FOXP3 were associated with interval detected tumors.

**Table 1:** The correlation between CD3, CD4, CD8, CD45, and FOXP3 counts compared with clinicopathologic features, Ki67, vessel invasion, CD163‐positive macrophages, elastosis, and detection mode (*n* = 282).

		CD3		CD4		CD8		CD45		FOXP3	
		Low	High	Low	High	Low	High	Low	High	Low	High
Variable	*N*	*N* (%)	*N* (%)	*N* (%)	*N* (%)	*N* (%)	*N* (%)	*N* (%)	*N* (%)	*N* (%)	*N* (%)
Histologic type											
NST	228	172 (81)	56 (81)	171 (81)	57 (81)	169 (80)	59 (84)	173 (82)	55 (79)	169 (80)	59 (84)
Other	54	41 (19)	13 (19)	41 (19)	13 (19)	43 (20)	11 (16)	55 (18)	15 (21)	43 (20)	11 (16)
		*p* = 0.94		*p* = 0.88		*p* = 0.40		*p* = 0.58		*p* = 0.40	
Tumor size (cm)											
<2	214	164 (77)	50 (72)	159 (75)	55 (79)	158 (75)	56 (80)	161 (76)	53 (76)	159 (75)	55 (79)
≥2	68	49 (23)	19 (28)	53 (25)	15 (21)	54 (25)	14 (20)	51 (24)	17 (24)	53 (25)	15 (21)
		*p* = 0.44		*p* = 0.97		*p* = 0.35		*p* = 0.97		*p* = 0.55	
Histologic grade											
1	76	64 (30)	12 (17)	61 (29)	15 (21)	60 (28)	16 (23)	62 (29)	14 (20)	64 (30)	12 (17)
2–3	208	149 (70)	57 (83)	151 (71)	55 (79)	157 (72)	54 (77)	155 (71)	56 (80)	148 (70)	58 (83)
		*p* = 0.04		*p* = 0.23		*p* = 0.37		*p* = 0.13		*p* = 0.03	
Lymph node status*											
Negative	187	145 (68)	42 (61)	147 (70)	40 (57)	140 (66)	47 (67)	139 (66)	48 (69)	146 (69)	41 (59)
Positive	94	67 (32)	27 (39)	64 (30)	30 (43)	71 (34)	23 (33)	72 (34)	22 (31)	66 (31)	28 (41)
		*p* = 0.25		*p* = 0.05		*p* = 0.90		*p* = 0.68		*p* = 0.15	
ER											
Positive	249	198 (93)	51 (74)	190 (90)	59 (84)	197 (93)	52 (74)	195 (92)	54 (77)	200 (94)	49 (70)
Negative	33	15 (7)	18 (26)	22 (10)	11 (16)	15 (7)	18 (26)	17 (8)	16 (23)	12 (6%)	21 (30)
		*p* < 0.001		*p* = 0.23		*p* < 0.001		*p* < 0.001		*p* < 0.001	
PR											
Positive	186	144 (68)	42 (61)	141 (67)	45 (64)	147 (69)	39 (56)	144 (68)	42 (60)	146 (69)	40 (57)
Negative	96	69 (32)	27 (39)	71 (33)	25 (36)	65 (31)	31 (44)	68 (32)	28 (40)	66 (31)	30 (43)
		*p* = 0.31		*p* = 0.73		*p* = 0.04		*p* = 0.23		*p* = 0.07	
HER2 status											
Negative	255	197 (93)	58 (84)	192 (91)	63 (90)	196 (93)	59 (84)	197 (93)	58 (83)	200 (94)	55 (79)
Positive	27	16 (7)	11 (16)	20 (9)	7 (10)	16 (7)	11 (16)	15 (7)	12 (17)	12 (6)	15 (21)
		*p* = 0.04		*p* = 0.89		*p* = 0.04		*p* = 0.01		*p* < 0.001	
Ki67											
Low	211	171 (80)	40 (58)	164 (77)	47 (63)	169 (80)	42 (60)	172 (81)	39 (56)	176 (83)	35 (50)
High	71	42 (20)	29 (42)	48 (23)	23 (33)	43 (20)	28 (40)	40 (19)	31 (44)	36 (17)	35 (50)
		*p* < 0.001		*p* = 0.09		*p* < 0.001		*p* < 0.001		*p* < 0.001	
LVI											
Negative	212	169 (80)	43 (62)	166 (78)	46 (66)	167 (79)	45 (64)	168 (79)	44 (63)	171 (81)	41 (59)
Positive	70	44 (20)	26 (38)	46 (22)	24 (34)	45 (21)	25 (36)	44 (21)	26 (37)	41 (19)	29 (41)
		*p* = 0.004		*p* = 0.035		*p* = 0.015		*p* = 0.006		*p* < 0.001	
BVI											
Negative	239	185 (87)	54 (78)	179 (84)	60 (86)	185 (87)	54 (77)	187 (88)	52 (74)	189 (89)	50 (71)
Positive	43	28 (13)	15 (22)	33 (16)	10 (14)	27 (13)	16 (23)	25 (12)	18 (26)	23 (11)	20 (29)
		*p* = 0.084		*p* = 0.796		*p* = 0.041		*p* = 0.005		*p* < 0.001	
CD163											
Low	212	182 (85)	30 (44)	168 (79)	44 (63)	178 (84)	34 (49)	178 (84)	34 (49)	180 (85)	32 (46)
High	70	31 (15)	39 (56)	44 (21)	26 (37)	34 (16)	36 (51)	34 (16)	36 (51)	32 (15)	38 (54)
		*p* < 0.001		*p* = 0.01		*p* < 0.001		*p* < 0.001		*p* < 0.001	
Elastosis											
Low	237	171 (80)	66 (96)	172 (81)	65 (93)	173 (82)	64 (91)	172 (81)	65 (93)	172 (81)	65 (93)
High	45	42 (20)	3 (4)	40 (19)	5 (7)	39 (27)	6 (9)	40 (19)	5 (7)	40 (19)	5 (7)
		*p* = 0.002		*p* = 0.02		*p* = 0.05		*p* = 0.02		*p* = 0.02	
Detection mode											
Screening	200	158 (74)	42 (61)	149 (70)	51 (73)	157 (74)	43 (61)	155 (73)	45 (64)	159 (75)	41 (59)
Interval	82	55 (26)	27 (39)	63 (30)	19 (27)	55 (26)	27 (39)	57 (27)	25 (36)	53 (25)	29 (41)
		*p* = 0.03		*p* = 0.68		*p* = 0.04		*p* = 0.16		*p* = 0.01	

*P* values were obtained using Pearson's chi‐square test. High CD3, CD4, CD 8, CD45, and FOXP3 counts are given by the upper quartile.

*n*, number of cases; NST, no special type.

* One case was excluded due to missing information on lymph node status.

When including all TIL subsets (CD45, CD3, CD4, CD8, and FOXP3) in multivariable logistic regression analyses as explanatory variables, and using histologic grade, ER/PR/HER2 status, Ki67, BVI, LVI, CD163 TAM, elastosis, and detection method as predicted (outcome) variables in separate regression models, we consistently found that FOXP3 independently predicted aggressive tumor features, such as ER negativity (odds ratio [OR] 4.4, 95% CI 1.7–11.6, *p* = 0.003), HER2 positivity (OR 4.1, 95% CI 1.5–11.5, *p* = 0.007), high Ki67 (OR 3.6, 95% CI 1.8–7.5, *p* = 0.001), BVI (OR 2.9, 95% CI 1.2–6.9, *p* = 0.015), LVI (OR 2.3, 95% CI 1.1–4.7, *p* = 0.025), and high CD163 TAM (OR 2.7, 95%CI 1.3–5.8, *p* = 0.009). In contrast, the other TIL subsets were not independently associated with these clinicopathologic features, except that, in addition to FOXP3, both CD3 and CD4 were independently associated with CD163 TAM status (OR 6.5, 95% CI 2.2–19.3, *p* = 0.001; and OR 0.3, 95% CI 0.1–0.7, *p* = 0.013).

### High levels of TIL subsets are associated with different categories of vascular invasion

High numbers of all TIL subsets were significantly associated with LVI. Also, high counts of CD45+ leukocytes as well as CD8+ and FOXP3+ lymphocytes were associated with BVI (Table 1).

Table 2 shows the association between levels of TILs and different combinations of vascular invasion: LVI−/BVI−, LVI+/BVI−, LVI−/BVI+, and LVI+/BVI+. All TIL subsets (except CD4) were significantly associated with these categories of vascular invasion, with FOXP3 displaying the strongest association (*p* < 0.001). The general pattern was that most LVI−/BVI− tumors (around 80%) had low numbers of infiltrating lymphocytes, whereas a much higher percentage (44–56%) of LVI+/BVI+ tumors were highly infiltrated (Table 2). LVI+/BVI− and LVI−/BVI+ tumors showed intermediate TIL percentages. Thus, both LVI and BVI associated with increased lymphocytic infiltration in tumor tissue.

**Table 2:** Counts of CD3, CD4, CD8, CD45, and FOXP3 TIL subtypes according to different categories of vascular invasion (*n* = 282).

	LVI−/BVI−	LVI+/BVI−	LVI−/BVI+	LVI+/BVI+	
	*n* (%)	*n* (%)	*n* (%)	*n* (%)	*P* value
Total	194 (68)	45 (16)	18 (6)	25 (10)	
CD3					
Low	155 (80)	30 (67)	14 (78)	14 (56)	0.028
High	39 (20)	15 (33)	4 (22)	11 (44)	
CD4					
Low	151 (78)	28 (62)	15 (83)	18 (72)	0.135
High	43 (22)	17 (38)	3 (17)	7 (28)	
CD8					
Low	154 (79)	31 (69)	13 (72)	14 (56)	0.05
High	40 (21)	14 (31)	5 (28)	11 (44)	
CD45					
Low	157 (91)	30 (67)	11 (61)	14 (56)	0.007
High	37 (19)	15 (33)	7 (39)	11 (44)	
FOXP3					
Low	159 (82)	30 (67)	12 (67)	11 (44)	<0.001
High	35 (18)	15 (33)	6 (33)	14 (56)	

Number of cases (*n*) and % within different categories of vessel invasion. *P* values were obtained using Pearson's chi‐square test. High CD3, CD4, CD8, CD45, and FOXP3 counts are given by the upper quartile.

### High levels of TIL subsets are associated with breast cancer molecular subgroups

There were 51% luminal A, 32% luminal B (HER2 negative), 6% luminal B (HER2 positive), 4% HER2‐type, and 7% triple‐negative tumors in this population‐based cohort (Table 3). High levels of all TIL subsets (except CD4) were significantly associated with breast cancer subtypes. Again, FOXP3 demonstrated the strongest association (*p* < 0.001). In general, luminal A and luminal B HER2‐negative tumors showed on average low‐grade lymphocytic infiltration in around 80% of tumors (Table 3 and supplementary material, Figure S1). In contrast, around 50% of HER2‐type and triple‐negative tumors showed high TIL counts. Intermediate values were found for luminal B HER2‐positive tumors (Table 3).

**Table 3:** Counts of CD3, CD4, CD8, CD45, and FOXP3 TIL subtypes by molecular subtype (*n* = 282).

	Luminal A	Luminal B		HER2 type	Triple negative	
		HER2−	HER2+			
	*n* (%)	*n* (%)	*n* (%)	*n* (%)	*n* (%)	*P* value
Total	145 (51)	89 (32)	18 (6)	9 (4)	21 (7)	
CD3						
High	29 (20)	19 (21)	6 (33)	5 (56)	10 (48)	0.009
Low	116 (80)	70 (79)	12 (67)	4 (44)	11 (52)	
CD4						
High	32 (22)	24 (27)	6 (33)	1 (11)	7 (33)	0.52
Low	113 (78)	65 (73)	12 (67)	8 (89)	14 (67)	
CD8						
High	28 (19)	20 (23)	6 (33)	5 (56)	11 (52)	0.002
Low	117 (81)	69 (77)	12 (67)	4 (44)	10 (48)	
CD45						
High	27 (19)	22 (25)	8 (44)	4 (44)	9 (43)	0.016
Low	118 (81)	67 (75)	10 (56)	5 (56)	12 (57)	
FOXP3						
High	24 (17)	19 (21)	9 (50)	6 (67)	12 (57)	<0.001
Low	121 (83)	70 (79)	9 (50)	3 (33)	9 (43)	

Number of cases (*n*) and % within molecular subtypes are given according to the St Gallen consensus criteria 2013 [23]. *P* values were obtained using Pearson's chi‐square test. High CD3, CD4, CD8, CD45, and FOXP3 counts are given by the upper quartile.

### Associations between TIL subsets and patient prognosis

The median follow‐up time was 138 months (range 108–168). Among the 282 patients, metastases or local recurrence were observed at follow‐up in 42 cases (15%), and 32 patients (11%) died of breast cancer.

**Figure 2:**
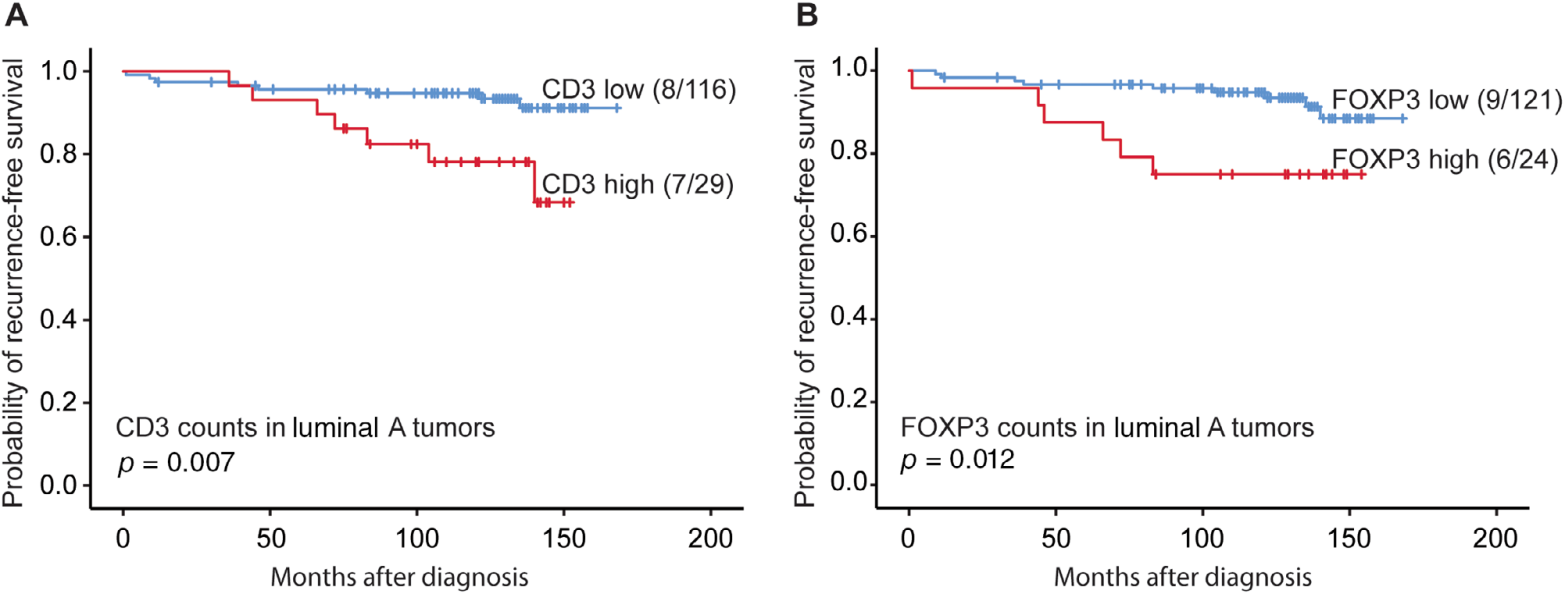
RFS in luminal A cases related to high (upper quartile) or low (A) CD3 and (B) FOXP3 counts. Survival curves were estimated using the Kaplan–Meier method (with log‐rank test for differences). For each category, number of events/total number of cases are given.

Univariate survival analysis of various TIL subsets across all breast cancer subtypes in the whole cohort did not reveal significant prognostic associations. Among luminal A tumors (*n* = 145), univariate survival analysis indicated that high levels (by upper quartile) of CD3‐ and FOXP3‐positive TILs were associated with reduced RFS (Figure 2), and FOXP3 was also associated with reduced DSS (*p* < 0.05).

High levels of both CD3 and FOXP3 were associated with reduced RFS in patients who had not received endocrine therapy (*p* = 0.018 and *p* = 0.028, respectively), but did not show significance in patients who received endocrine therapy. Also, high levels of CD3 and CD4 were associated with reduced RFS in patients who did not undergo chemotherapy (*p* = 0.011 and *p* = 0.032, respectively), while there was no significant difference in cases with treatment. As expected, treated and untreated cases (for both endocrine treatment and chemotherapy) showed significant differences with respect to tumor diameter, histologic grade, lymph node status, as well as ER and PR positivity.

## Discussion

Breast tumors are often infiltrated by a diverse population of immune cells. The composition of TIL subsets and their interaction with tumor cells might result in either tumor regression or progression [26]. Our present study indicates that different TIL subpopulations are strongly related to more aggressive breast cancer subgroups such as ER‐negative or HER2‐positive tumors, and cases with high proliferation, as assessed by Ki67 levels. Notably, among the TIL subsets, FOXP3 represented an independent marker for prediction of aggressive tumor subgroups in this series. These findings support the notion that FOXP3 is a master regulator in the TIME [27]. Our data are consistent with other studies, indicating that composition of the immune cell infiltrates represents a fundamental part of breast cancer progression [14, 26, 28, 29, 30, 31]. However, less is known about how immune cells associate with other components of the tumor stroma.

Here, we found that different TIL subsets were strongly associated with TAMs and vascular invasion. Importantly, we demonstrated that tumors with high‐grade inflammation showed increased frequency of both LVI and BVI. Vascular invasion has been recognized as a crucial step in metastasis and may indicate disseminated disease and unfavorable prognosis among cancer patients [32]. Both TILs and TAMs have important roles in neoplasia and produce angiogenic growth factors and cytokines that potentiate tumor angiogenesis and progression [33, 34, 35, 36, 37, 38]. In line with this, TGF‐β produced by macrophages and regulatory T cells (FOXP3+) can promote epithelial–mesenchymal transition in breast tumor cells and thereby enhance tumor cell motility and intravasation [39, 40]. This may support our findings of a strong relationship between high TIL content and vascular invasion. In particular, FOXP3‐positive lymphoid cells were strongly associated with both LVI and BVI.

We previously demonstrated that there is a strong association between stromal elastosis and screen‐detected cancers [11]. Notably, the present study indicates that high levels of TIL subsets are strongly inversely associated with tumor elastosis, and high counts of CD3+, CD8+, and FOXP3+ lymphocyte subsets are also related to interval detected cancers. To the best of our knowledge, this is the first integrated study of the breast cancer microenvironment in relation to tumor detection mode, and with particular attention to lymphoid cell subtypes, stromal elastosis, and presence of vascular invasion.

Interval detected breast cancers are more often basal‐like and HER2 positive [41] and demonstrate a stiffer collagen stroma and higher immune cell infiltration [42] in contrast to screen‐detected tumors. An absence of elastosis and higher immune cell content may stimulate a more angiogenic tumor microenvironment with increased risk of vascular invasion and tumor spread [43, 44].

In the luminal A subtype, high numbers of CD3+ and FOXP3+ cells were associated with reduced RFS, and FOXP3+ cells from the tumor margins were also related to reduced DSS. In general, lower numbers of CD3+ lymphocytes are usually associated with poorer prognosis due to a suppressed immune response, and FOXP3 is known to suppress antitumor responses and facilitate cancer progression [45]. In contrast, higher CD3+ lymphocyte numbers were associated with reduced RFS in our study. Although our findings on FOXP3 are consistent with previous work [46, 47, 48], the data on CD3 might be controversial. Still, our observations are in line with a recent report in which the authors found that high T lymphocyte and Treg infiltration in primary breast cancer correlated with poor prognostic factors and shorter survival [49].

The role of TILs, especially focusing on CD8 and FOXP3 as prognostic markers within molecular subtypes, has been evaluated in several studies, with particular attention paid to HER2‐positive and triple‐negative breast cancer patients [50, 51, 52, 53, 54, 55, 56, 57, 58]. In contrast, luminal tumors have been less well studied. Recently, a few publications have focused on luminal HER2‐negative tumors, without finding significant prognostic signals related to TILs [18, 59], although the methods used are somewhat different from our study. These reports presented the total number of TILs in HE‐stained sections, without differentiating between TIL subsets. In contrast, a meta‐analysis from 2017 suggested that high FOXP3 levels predicted a poorer prognosis in ER‐positive breast cancer [60], and this observation is in agreement with our findings of reduced survival associated with high levels of FOXP3 within the luminal A subgroup. Furthermore, a study from 2019 examined TIL composition and the phosphatidylinositol 3‐kinase pathway in luminal breast cancer. It was suggested that high levels of CD8+ lymphocytes were observed in *PIK3CA*‐mutated tumors, and that these patients were more likely to experience disease recurrence [61]. Further validation of the prognostic value of TIL subsets is needed to improve prognostication and potential treatment stratification, and to avoid overtreatment in luminal breast cancer.

Although several previous studies have focused on the significance of TIL subsets in breast cancer, methods for evaluation were different among these investigations [62, 63]. Standardization of methods for scoring of TILs is still a key issue. A limitation in our study was the measurement of TILs (CD3, CD4, CD8, CD45, and FOXP3) and macrophages (CD163) by immunohistochemistry on TMA sections. Using TMAs might underestimate the levels due to tumor heterogeneity and limited sampling. To reduce this potential confounding factor, three cores were extracted from the invasive edge of the tumor (diameter 1.0 mm). Furthermore, immunohistochemical staining of CD4 might also be positive on macrophages and dendritic cells, potentially influencing CD4 cell counts, although cell morphology was assessed during counting to minimize this bias.

In summary, our study on breast cancer indicates associations between high levels of different TIL subtypes and TAM levels, vascular invasion (LVI and BVI), absence of stromal elastosis, tumor cell proliferation, and interval detection mode. Our findings suggest an importance of tumor microenvironment factors, and interactions between them, for progression of breast cancer subgroups. Notably, the presence of FOXP3+ lymphoid cells was strongly associated with most factors studied, and was also prognostic within the low‐grade luminal A category. The interplay between cancer cells and their microenvironment to influence cancer progression is a challenging and extremely complicated process, which needs further investigation.

## Author contributions statement

YC, TK and LAA contributed to study design, data interpretation, literature search and writing of the manuscript. YC, TAK and HA participated in data collection. YC, TAK and EW performed data analysis and generation of figures. All authors approved the final version of the manuscript.

## Supporting information

**Figure S1.** Median CD3, CD4, CD8, FOXP3, and CD45 counts in molecular breast cancer subgroups
**Table S1.** Counts for different TIL categories
**Table S2.** Cross‐correlation between different TIL subsets and tissue macrophages
