# Supplementary material for: Tumor‐associated lymphocytes and macrophages are related to stromal elastosis and vascular invasion in breast cancer
Source: J Pathol Clin Res. 2021 Jun 2;7(5):517–27. doi: 10.1002/cjp2.226 (PMC8363927; doi:10.1002/cjp2.226)
Supplement: Supplementary file 1 — Figure S1. Median CD3, CD4, CD8, FOXP3, and CD45 counts in molecular breast cancer subgroups Table S1. Counts for different TIL categories Table S2. Cross‐correlation between different TIL subsets and tissue macrophages [file CJP2-7-517-s001.docx]

**Tumor-associated lymphocytes and macrophages are related to stromal elastosis and vascular invasion in breast cancer**

Chen *et al*. *J Pathol Clin Res* DOI: 10.1002/cjp2.226

**Supplementary Material**

**Figure S1.** Median CD3, CD4, CD8, FOXP3 and CD45 counts in molecular breast cancer subgroups

**Table S1.** Counts for different TIL categories

**Table S2.** Cross-correlation between different TIL subsets and tissue macrophages

**
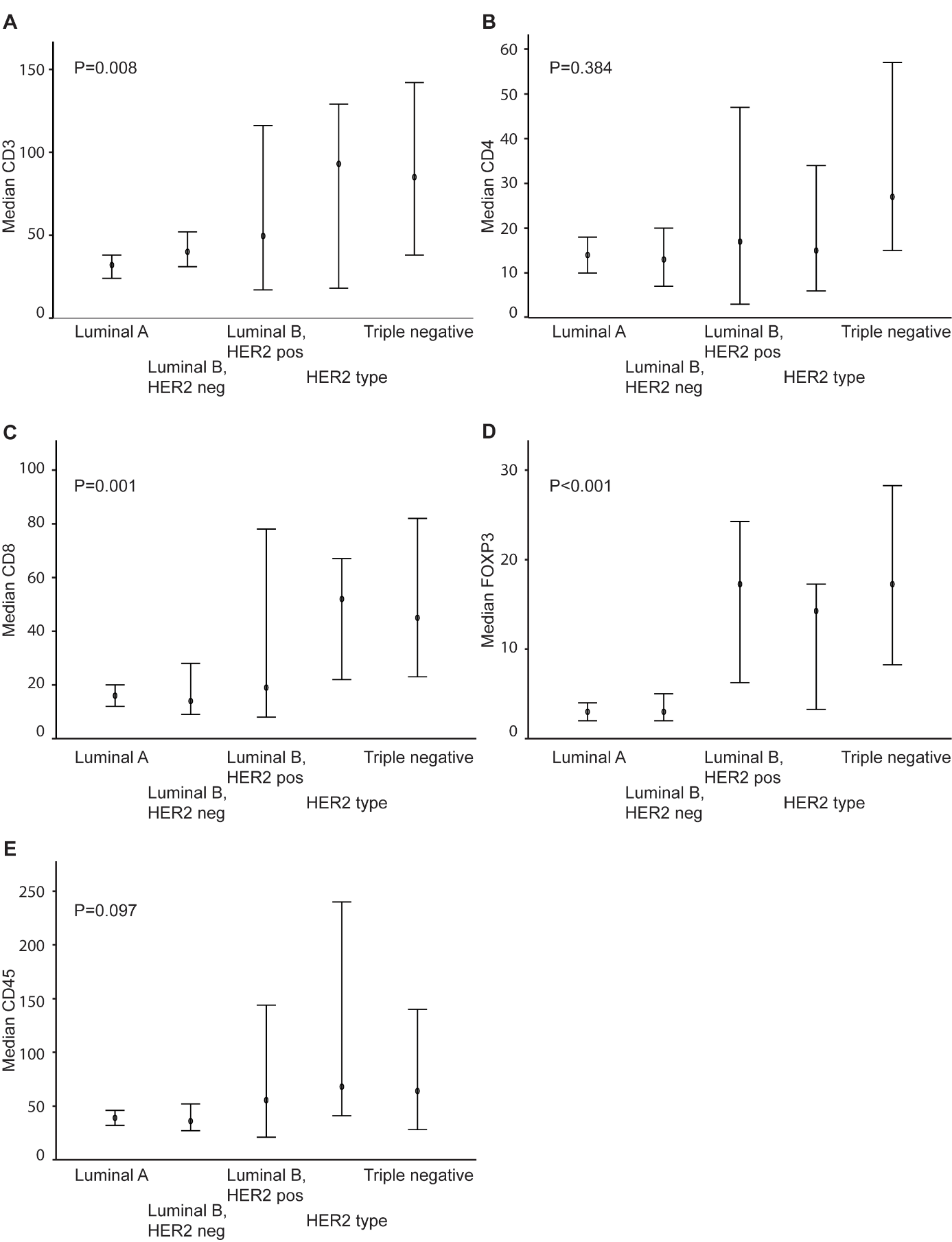
**

**Figure S1. Median (A) CD3, (B) CD4, (C) CD8, (D) FOXP3 and (E) CD45 counts in molecular breast cancer subgroups.**

Data represent median values with error bars denoting the limits of the 95% confidence intervals.

**Table S1.** Counts for different TIL categories (10 x 10 grid-lines; 0.31 x 0.31 mm; total 0.096 mm^2^) (n=282)

|  | **Median** | **Range** | **Mean** |
| --- | --- | --- | --- |
| **CD45** | 42 | 1-498 | 74 |
| **CD3** | 37 | 0-321 | 59 |
| **CD4** | 15 | 0-209 | 28 |
| **CD8** | 18 | 0-216 | 33 |
| **FOXP3** | 4 | 0-156 | 9 |

**Table S2.** Cross-correlation between different TIL subsets and tissue macrophages

| **Variables** | **CD45+ TIL** | **CD3+ TIL** | **CD4+ TIL** | **CD8+ TIL** | **FOXP3+ TIL** | **CD163+ TAM** |
| --- | --- | --- | --- | --- | --- | --- |
| **CD45+ TIL** | 1 |  |  |  |  |  |
| **CD3+ TIL** | 0.73** | 1 |  |  |  |  |
| **CD4+ TIL** | 0.63** | 0.78** | 1 |  |  |  |
| **CD8+ TIL** | 0.72** | 0.67** | 0.58** | 1 |  |  |
| **FOXP3+ TIL** | 0.38** | 0.50** | 0.46** | 0.48** | 1 |  |
| **CD163+ TAM** | 0.43** | 0.52** | 0.29** | 0.41** | 0.30** | 1 |
| Note: *N*=282. Spearman`s correlation coefficients are reported (***p* < 0.01) | | | | | | |
